# Structure-Controlled Porous Cordierite Ceramics with High Solid Content Prepared by Pickering Emulsion Technique Using Sucrose as a Porogen

**DOI:** 10.3390/ma15093410

**Published:** 2022-05-09

**Authors:** Xuezhu Luan, Jinhong Li, Wuwei Feng, Rui Liu, Shuo Liu, Ziyao Wang

**Affiliations:** 1 Institute of Innovative Science and Technology, Shenyang University, Shenyang 110044, China; ziyao46@163.com; 2 College of Mechanical Engineering, Shenyang University, Shenyang 110044, China; 3 Beijing Key Laboratory of Materials Utilization of Nonmetallic Minerals and Solid Wastes, National Laboratory of Mineral Materials, School of Materials Science and Technology, China University of Geosciences, Beijing 100083, China; jinhong@cugb.edu.cn (J.L.); wfeng@cugb.edu.cn (W.F.); ls112233@163.com (S.L.); 4 State Grid Liaoning Electric Power Co., Ltd., Shenyang 110044, China; liur0815@163.com

**Keywords:** cordierite, pickering emulsion, sucrose, high solid content, compressive strength

## Abstract

Porous cordierite ceramics (PCCs) with stable 3D microstructures were prepared by Pickering emulsion technique using sucrose as a porogen. The microstructural and mechanical properties could be adjusted by varying O/S ratios, sintering temperature, and sucrose content. The formation of the spherical structure was due to the broken oil bubbles. The appearance of cordierite and the concurrent consumption of sucrose were responsible for the observation of gradient pore structure. When the O/S ratio was 2, the pore-structure-controlled PCCs with cordierite as the main phase was obtained after sintering at 1300 °C. With the addition of 30 wt.% of sucrose, the obtained PCCs possessed high solid content of 45 vol.%, the porosity of 90.83%, the compressive strength of 6.09 MPa, and the optimized thermal conductivity of 0.4794 W/m.K. Compared with the predecessors’ research results, the as-prepared precursor of PCCs with sucrose content had the lowest initial Zeta potential without adjusting the pH to ensure the stable suspension. Our results showed that the addition of sucrose not only acts as a solvent to increase the solid content, but also acts as a pH modifier to maintain precursor stability, which enables the increase in compressive strength. In this work, via the scenario of “oil droplet” 3D accumulation, the stable and orderly spatial arrangement of the micro-emulsion system was successfully realized to obtain the structure-controlled PCCs by controlling the precursor conditions.

## 1. Introduction

**Figure 1:**
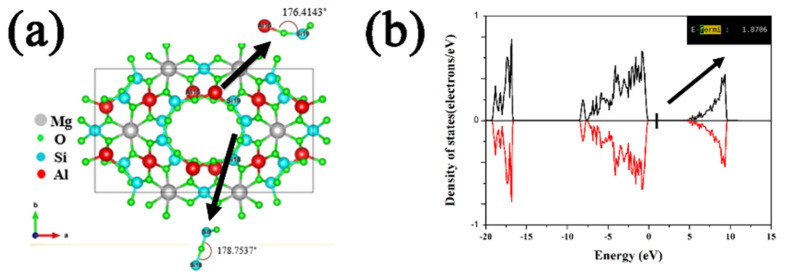
(**a**) Chemical bonds in the crystal structure of cordierite, (**b**) The DOS diagram of cordierite.

Porous ceramic material is a kind of unique material with high porosity prepared by a special sintering process [1,2]. Porous ceramic material has a controllable pore structure, high open porosity, long service life, and good product regeneration performance. It also possesses anti-corrosion of organic media and good biological inertia [3,4,5,6,7]. Due to the above-mentioned properties, it has been widely used in various industrial fields, such as liquid/gas filters, heat insulation, catalytic carriers, gas distributors, membrane separation, and pollution control [5,6,7]. Cordierite (Mg_2_Al_4_Si_5_O_18_), as a traditional ceramic material, has received extensive attention due to its excellent thermal expansion coefficient [8,9,10]. Cordierite has a particular structure with Mg atoms hosted in the skeleton gap between [AlO_4_] and [SiO_4_] tetrahedron, as shown in Figure 1a [11], in which the approximate 180° bond angle between Si-Al and Si-Si is response for the low thermal expansion for cordierite. The energy gap is analyzed from the DOS diagram (Figure 1b) and the Fermi level is in the range of zero DOS value, indicating the low conductivity of the cordierite. Therefore, cordierite is widely used in high-temperature applications such as catalyst carriers, high temperature soot filtration, and so on [12,13,14]. From the viewpoint of applications, it is desirable to develop cordierite ceramics with high porosity and spherical, open, and inter-connected pore structure [11,12,15]. Since pore structure and size distribution could significantly influence the effective thermal conductivity [16,17,18,19,20,21,22,23]. Qiu et al. [17] have explored the relationship between different pore sizes and thermal conductivity, and found that the optimization of reduced pore size is beneficial to reducing effective thermal conductivity. It was also found that optimizing pore size distribution exerts a significant impact on properties [24]. Han et al. [21] prepared porous CaAl_2_Si_2_O_8_ ceramics by the foam-gel-casting method, and used some organic additives to optimize the pore size to form a 3D network structure. However, thus-prepared ceramics are of uneven pore size and poor mechanical strength. A large number of organic additives release greenhouse gases and toxic gases during the sintering process and thus cause pollution [25,26,27]. Recently, the Pickering emulsion technique has been widely studied because of its simple preparation process for controlled porous structures [28,29,30,31,32]. Ma et al. [33] synthesized cordierite with a pore size ranging from 40 to 150 μm using this method. Wang et al. [34] prepared 3D hydroxyapatite ceramics with pore sizes ranging from 3 to 5 μm. Li et al. [35,36] used different amounts of sucrose as additives to adjust the pore structure and obtained TiO_2_ ceramic with a pore size of 3–18 μm. Therefore, the Pickering emulsion technique is adequate for controlling the highly ordered porous microstructure. In the previous study, Luan et al. [37] used particle-stabilized emulsions to prepare hierarchically porous cordierite ceramics with a solid content of 40 vol.% by introducing starch as a modifier, showing its unique structural and performance advantages. However, the addition of starch played a positive role in the formation of the hierarchically porous structure, but also decreased the solid content of the system. In order to increase the solid content of PCCs, which realized the orderly arrangement of oil droplets in three-dimensions in order to improve the compressive strength, sucrose was used as a solvent in this study.

In this experiment, PCCs were prepared by the Pickering emulsion technique. Pure materials were introduced to prepare stable cordierite emulsion precursors. Sucrose was used as a porogen to optimize pore size distribution. The pore characteristics and phase composition were essential to improving the thermal and mechanical properties [24]. The relationship between porosity and compressive strength was balanced by adjusting the pore structure via changes in the sintering temperature, sucrose, and octane content. The mechanism for the formation of the facilities was also discussed.

## 2. Experiments

### 2.1. Materials and Preparation

**Figure 2:**
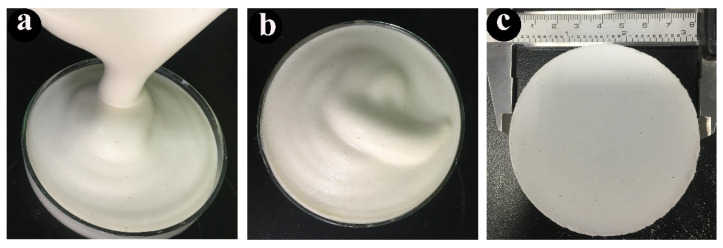
The morphological changes of PCCs (**a**) Forming stage, (**b**) Drying stage, (**c**) Sintering stage.

Commercially available magnesia, alumina, and silica (AR, Shanghai Xinding Metallurgical Materials Co., Ltd., Shanghai, China) were mixed in a stoichiometric ratio to obtain a ceramic suspension. The mixture containing 2.0 wt.% ammonium polyacrylate salt (Adamas Reagent Co., Ltd., Shanghai, China), 0.5 wt.% PVA (AR, Adamas Reagent Co., Ltd., Shanghai, China) and different additive amount of sucrose (10–40 wt.%) (Beijing Chemical Works, Beijing, China) with 0.15 mol/L CaCl_2_, was ball-milled with ZrO_2_ balls (10 mm in diameter) at full speed of 800 rpm for 24 h. The solid content was firstly determined at 40 vol.%. 2.0 wt.% propyl gallate (C_10_H_12_O_5_) (AR, Adamas Reagent Co., Ltd., Shanghai, China) was dissolved in ethanol (AR, Beijing Chemical Works, Beijing, China) and then transferred to the suspension to in situ modify the surface properties of particles to be partially hydrophobic, facilitating their adsorption at the oil–water interface. Different concentrations of octane (100–300 vol.%) (AR, Beijing Chemical Works, Beijing, China) were added to the suspended mixture, which was stirred for 5 min by an electric blender (HR1613, Philips, Amsterdam, The Netherlands) at full speed of 16,000 rpm. Afterward, the emulsion was transferred to the culture vessels and dried at room temperature for two weeks. Finally, the obtained samples were calcined at high temperatures (1100–1400 °C). The morphological changes of PCCs at the different stages are shown in Figure 2.

### 2.2. Characterization

The phase composition of the samples was characterized by X-ray powder diffraction (XRD, D’Max-Ra12 kW, Ouyatu, Tokyo, Japan). The morphology was analyzed by scanning electron microscope (SEM, S4800, Hitachi, Tokyo, Japan) after spraying gold particles on the surface of the samples. The porosity of the samples was measured based on Archimedes’ principle according to Equation (1):

P = (M_1_/M_2_ − M_3_) × ρ_1_ (1)

M_1_ = Dry weight of samples placed in the drying oven for 24 h.

M_2_ = Wet weight of samples complete immersion in distilled water under constant vacuum.

M_3_ = Float weight of samples in water.

ρ_1_ = Density of distilled water (1 g/cm^3^).

P = Porosity.

Six samples from each group were measured to obtain reliable porosity. The Pore size distribution of the samples was evaluated by mercury intrusion porosimeter (PoreMasterGT60, Quantachrome, Boynton Beach, FL, USA).

The compressive strength of calcined samples was determined by a universal testing machine (MTS Systems Co., Ltd., Beijing, China) at 0.05 mm/min propulsion speed at room temperature, and each test was repeated six times to obtain credible results.

## 3. Results and Discussion

### 3.1. Properties of PCCs Emulsions

**Figure 3:**
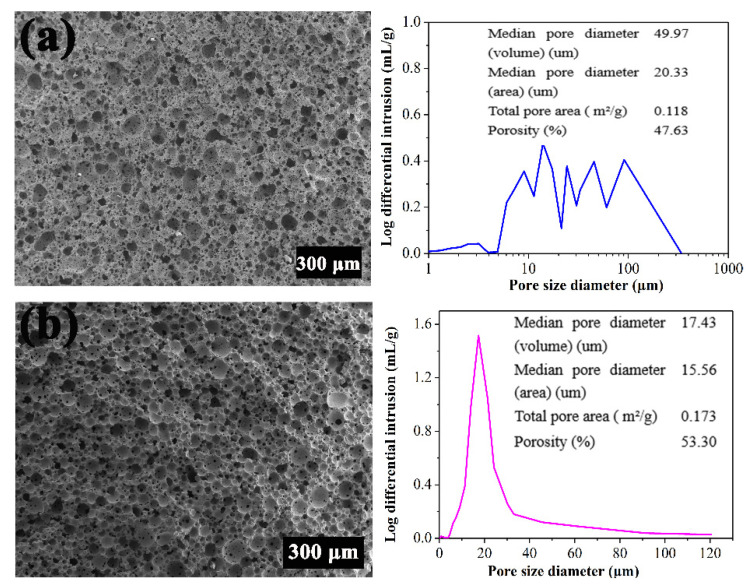
SEM images and pore size distribution of PCCs prepared by different methods (**a**) direct foaming method, (**b**) Pickering emulsion technique.

At present, porous ceramics are mainly obtained by the traditional preparation process [33], which cannot effectively control the structure to meet the requirements of various industries. Recently, the Pickering emulsion technique has attracted a great deal of interest in manufacturing of high porosity ceramics [34]. As shown in Figure 3a, PCCs with complex and non-uniform pore structure and large pore size spanning were prepared by a direct foaming method. The pore size distribution of PCC_S_ demonstrated many prominent peaks ranging from 10 μm to 100 μm, with an average pore diameter of 49.97 μm and a porosity of 47.63%. However, in Figure 3b, PCCs prepared by the Pickering emulsion technique exhibited an average pore diameter of 15.56 μm, with a porosity of 53.30%. The tiny pores result in a larger surface area, which is advantageous for catalyst support and adsorption. The mechanical properties of PCCs mainly depend on the interaction between their internal pore and cells. The Pickering emulsion technique facilitates the stability of particles at the interface during drying and sintering. PCCs with highly ordered pores reduced the pore size and increased the porosity, which effectively improved physical properties. Therefore, with facile preparation processes, the Pickering emulsion technique can control the porous structure, and has potential application prospects in preparing porous ceramics.

### 3.2. Effects of Oil Contents on the Morphology of PCCs

In this experiment, the surface of the particles was modified with propyl gallate to obtain stable fluid interface capability, which stabilizes the particles at the oil–water interface for a long time. When the suspension is emulsified, the hydrophilic portion of the particles tends to enter the aqueous phase, while the hydrophobic portion tends to enter the oil phase. Therefore, the precursors are stably arranged on the surface of the oil droplets, resulting in a spherical macro-porous structure.

**Figure 4:**
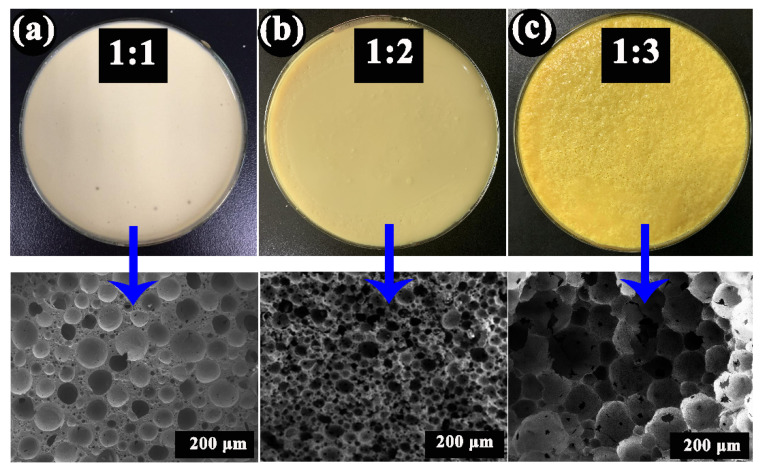
The microscopic pore structure of PCCs with different O/S ratio (**a**) 1:1, (**b**) 2:1, (**c**) 3:1.

As shown in Figure 4, the oil content significantly affects the microscopic morphology and the microstructure. At the O/S ratio of 1, the pore size distribution was uneven with a low porosity. With the increase in oil content, the porosity increased gradually, and the pore size distribution tended to be uniform. At the O/S ratio of 2, the pore structure of PCCs was uniform with small pore size and high opening porosity. As shown in Figure 4c, when the O/S ratio was 3, the thinning film of the particles distributed at the oil–water interface was not strong enough to keep the abundant adjacent oil droplets separate from each other. Therefore, the adjacent oil droplets merged and grew to form larger pore sizes. We can control the pore structure by selecting different O/S ratios. In order to obtain PCCs with high porosity, the balance of the precursor to the oil droplet volume needed to be adjusted, and control the properties of solvents to make the oil droplets uniformly and stably dispersed. It was optimized in our work that the light PCCs with uniform and 3D connected pore structure and high porosity can be obtained when the O/S ratio is 2.

### 3.3. Effects of Sucrose Content on the Morphology and Property of PCCs

By changing the properties of particles or solvents in the dispersion system, Pickering emulsion with high solid content can be obtained. Pickering emulsion contains aqueous and organic phases, and a large number of surfactants are added as stabilizers to maintain the system’s stability. Many colloidal particles can be used as stabilizers to obtain ultra-high stability due to the irreversible adsorption of colloidal particles at the interface. Therefore, high solid content is the key to obtaining a stable porous structure.

**Figure 5:**
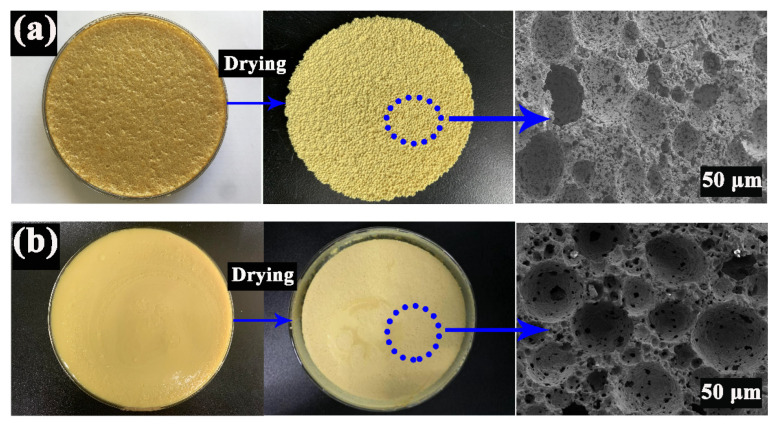
Pore structure of PCCs with (**a**) 20 vol.% and (**b**) 40 vol.% solid content, respectively.

**Figure 6:**
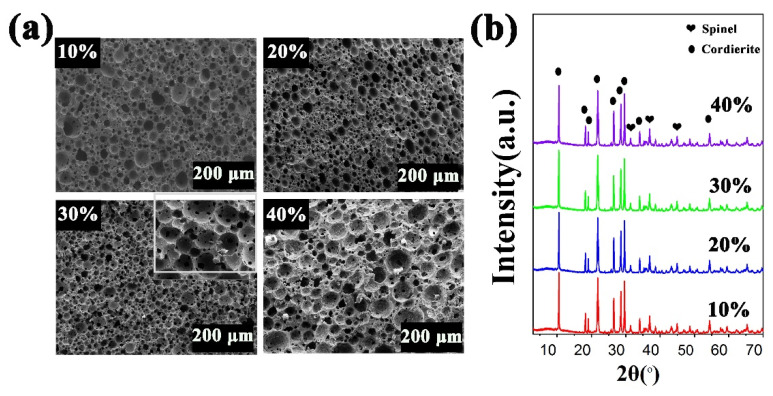
SEM images (**a**) and, XRD results (**b**) of PCCs with different sucrose content.

**Figure 7:**
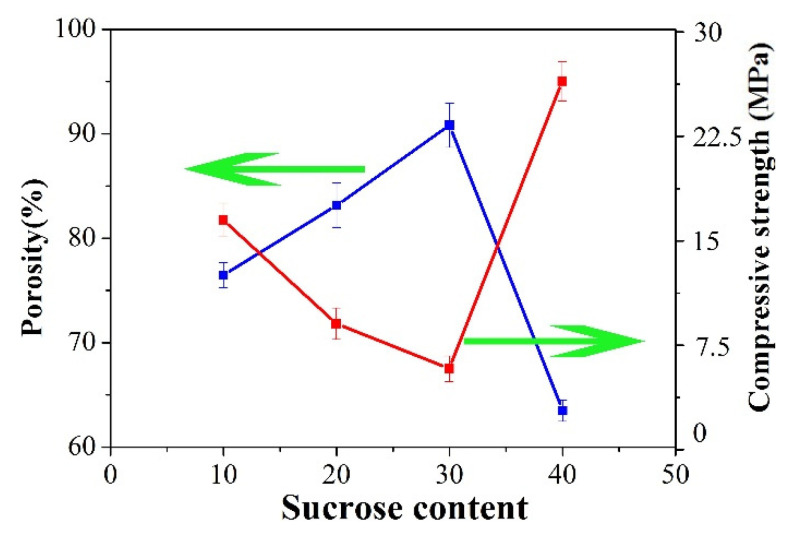
Porosity and compressive strength of the prepared PCCs sintered with different sucrose content at 1300 °C for 2 h.

As shown in Figure 5a, PCCs with low solid content had periodic microstructure and loose arrangement. With increasing the solid content, the pore structure gradually exhibited a uniform spherical shape with a reduced pore size (Figure 5b). Ma et al. [33] prepared cordierite ceramics with 30 vol.% solid content. Wang et al. [34] prepared 3D hydroxyapatite ceramics with 25 vol.% solid content. Li et al. [38] prepared Al_2_O_3_ ceramics with 40 vol.% solid content using sucrose as an additive. In this experiment, PCCs with solid content of 45 vol.% were obtained by using different content of sucrose additive after sintering at 1300 °C for 2 h. It was worth mentioning that sucrose would not affect the formation of the cordierite phase during the sintering process (Figure 6b). The melted sucrose not only acts as a solvent to increase solid content, but also acts as a pore-forming agent to increase porosity during high-temperature sintering. As shown in Figure 6a and Figure 7, when the sucrose content was 10 wt.%, PCCs showed dense spherical pores with a porosity of 76.47% due to the high solid content. As the sucrose content increased to 30 wt.%, the porosity of PCCs increased and the pore size became smaller. The pore structure could be modified by the addition of sucrose, which was evidenced by the internal pore-window structure in Figure 6a. When the sucrose content was 40 wt.%, the viscosity of the suspension was too high to promote the foaming process, causing the deformation of the green structure, resulting in reduced porosity of 63.49%. Due to the combination of porous microstructures, the mechanical strength of PCCs with sucrose addition was still high [39] (Figure 7). With 30 wt.% sucrose, PCCs with 90.83% of porosity, 6.09 MPa of compressive strength, and uniform pore size distribution can be obtained.

### 3.4. Effects of Sucrose on the Stability of PCCs Emulsions

**Figure 8:**
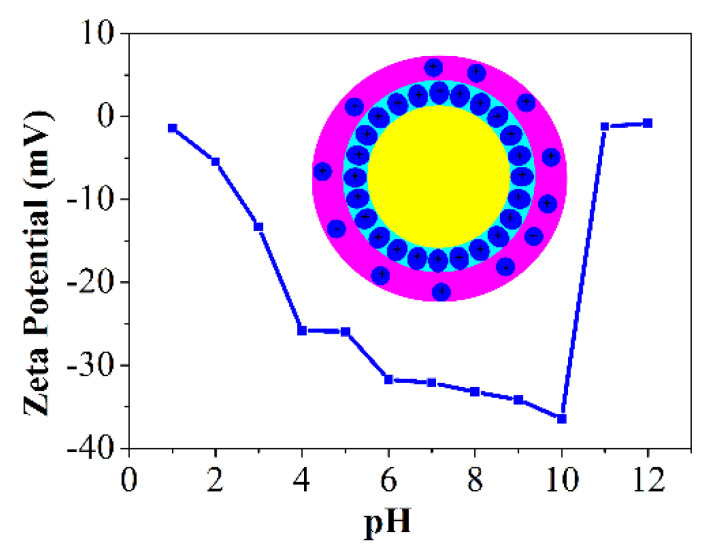
Zeta potential of PCCs suspension with different pH values.

As for the Pickering emulsion technique, the properties of suspension will change significantly when applied with a small external force, for example, leading to the aggregation of suspension particles to lower the energy of the system [33,34,35,36]. The stability of suspension mainly depends on the interaction between colloidal particles. Therefore, it is necessary to modify the interaction between colloidal particles to obtain a stable suspension. Zeta potential is an essential factor affecting the stability of the emulsion, and it also determines whether the colloidal particles are stable or inclined to flocculate. When the colloidal particles have a larger surface potential, the van der Waals force can be overcome, thus exhibiting a repulsive state. The particles can be dispersed stably in the suspension. When the colloidal particles have a small surface potential, the van der Waals force between the colloidal particles plays a major role, and cause the particles tend to agglomerate in the suspension. Usually, the addition of acid or alkali can change the surface potential of colloidal particles to regulate the distribution of colloidal particles in the suspension. In the previous study, Luan et al. [37] prepared porous ceramics with the emulsion pH value of 11, which was adjusted using ammonia and hydrochloric acid to obtain a stable ceramic suspension. As shown in Figure 8, when the pH value of the dispersion system ranges from 9 to 10, the lowest surface potential of the particles leads to the dominant repulsive force in the system. Therefore, the colloidal particles tend to be uniformly dispersed. In this experiment, the emulsion with 30% sucrose content had an incipient pH value of 9.8, which was suitable for preparing a highly stable emulsion. Therefore, no additional acid or alkali is needed to adjust the pH value to obtain a stable emulsion, which is a highlight of this work.

### 3.5. Effects of Temperature on the Morphology of PCCs with 30% Sucrose Content

**Figure 9:**
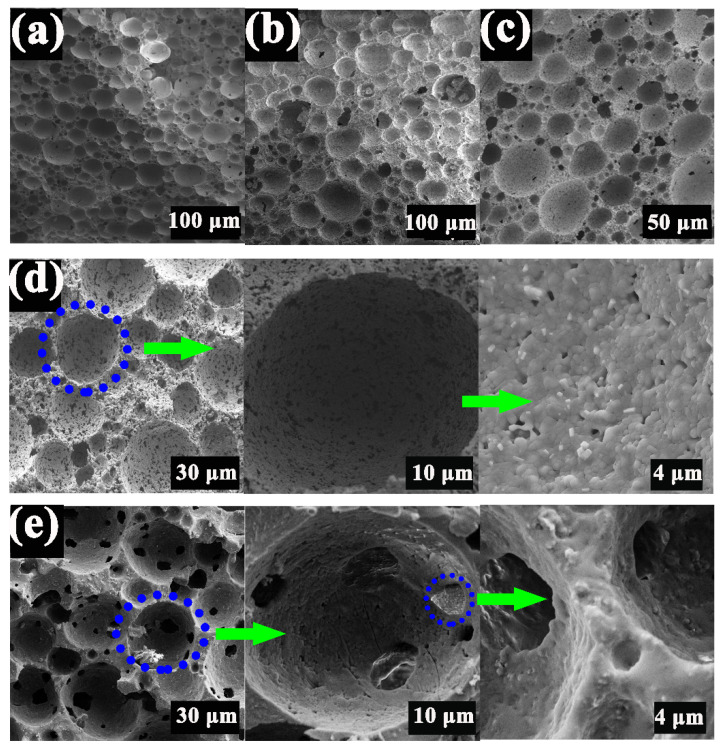
SEM images of PCCs with 30 wt.% sucrose content sintered at different temperature for 2 h (**a**) 25 °C, (**b**) 1100 °C, (**c**) 1200 °C, (**d**) 1300 °C, (**e**) 1400 °C.

**Figure 10:**
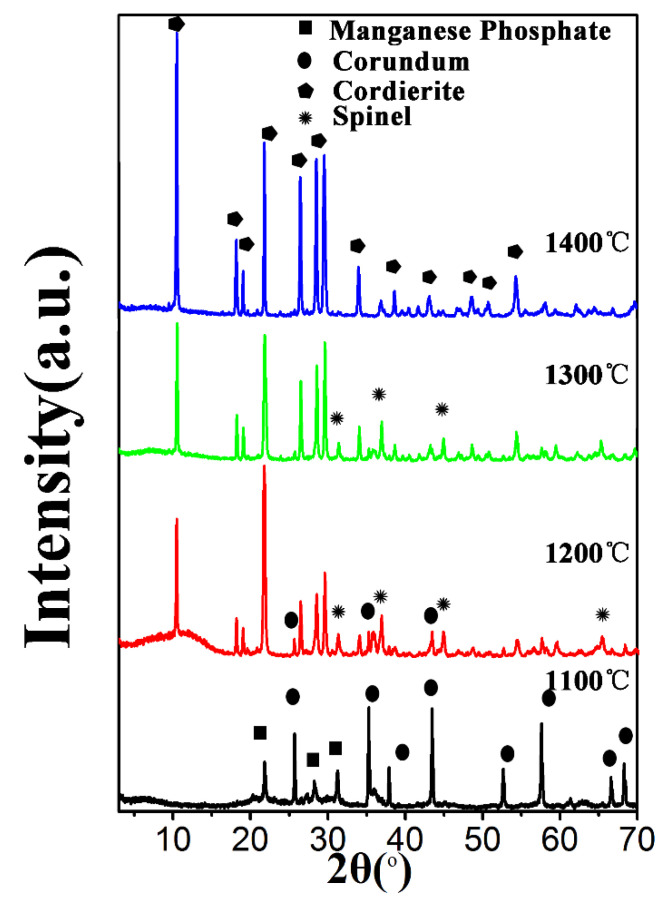
XRD results of the PCCs with 30 wt.% sucrose content sintered at different temperatures.

Figure 9 shows the representative microcosmic morphology of PCCs sintered at different temperatures from 1000 °C to 1400 °C for 2 h. It can be seen that PCCs were all of spherical shape, and the green body without sintering was composed of closed cells (Figure 9a). With increasing sintering temperature from 1100 °C to 1300 °C, the PCCs formed a 3D-ordered porous structure with significantly increased porosity of open cells. When the temperature was raised to 1400 °C, the adjacent pores were in contact during the sintering process, and the wall between the pores was broken to form interconnected macropores, resulting in the decrease in porosity and an increase in pore size, accompanied by the uneven pore size distribution. Figure 10 shows the XRD spectrum of PCCs prepared at different temperatures. The main phases were corundum and manganese phosphate when sintered at 1100 °C, and no cordierite phase appeared. When sintered at 1200 °C, spinel and cordierite began to appear. The main peak of cordierite was clearly visible when sintered at 1300 °C and became wider, stronger, and sharper when sintered at 1400 °C. It can be seen that cordierite is the main crystal phase in the 1400 °C sintered sample, analogous to the case sintered at 1300 °C. In order to control the final architectural connectivity and porosity of PCCs, 1300 °C was chosen as the optimum sintering temperature.

The addition of sucrose adds multiple important functions, acting as a pore-forming agent, solvent, and binder to increase the solid content, and also prompting a large number of colloidal particles to adhere to the surface of the O/W droplets, resulting in the improvement of the stability of PCCs. When the sucrose content was 30 wt.%, a large number of particles were distributed on the walls of the spherical macropore to form a uniform microstructure, which was conducive to increasing the specific surface area.

### 3.6. Effects of Sucrose Content on the Thermal Conductivity of PCCs

**Figure 11:**
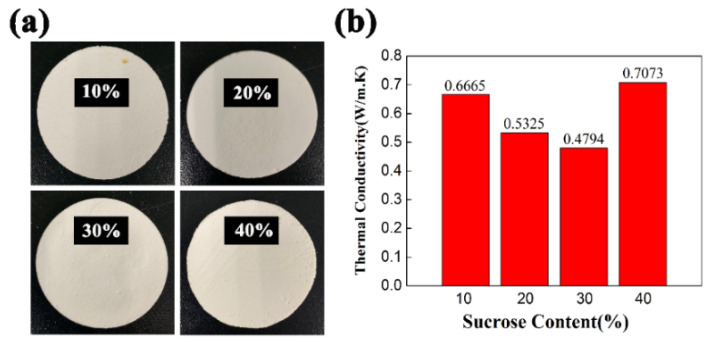
Digital photographs (**a**) and the thermal conductivities (**b**) of PCCs sintered with different sucrose content at 1300 °C for 2 h.

With the development of science and technology, it is particularly important to determine the thermal conductivity for the development of new materials. In this study, in addition to optimizing the preparation process to improve the structure and mechanical properties, it is also important to investigate the thermal properties of PCCs. The effect of sucrose content on the thermal conductivity of PCCs is shown in Figure 11. The thermal conductivities of PCCs with 10 wt.% sucrose content was 0.6665 W/m.K. With the increase in sucrose content from 10 to 30 wt.%, the thermal conductivity of PCCs decreased to 0.4794 W/m.K, which indicated that the pore size had a great influence on the thermal conductivity of PCCs. The introduction of sucrose into PCCs is beneficial to improving the solid content of the slurry and avoiding the combination of bubbles, leading to a uniform pore size distribution, which results in a decrease in thermal conductivity. When the sucrose content increased further to 40 wt.%, the thermal conductivity increased to 0.7073 W/m.K. due to the destruction of the pore structure. The addition of sucrose into the uniform microstructure of PCCs tended to reduce the thermal conductivity. With a sucrose content of 30 wt.%, the pore size was significantly reduced, which optimized the thermal conductivity of PCCs. The optimal PCCs containing 30 wt.% sucrose possessed porosity of 90.83%, compressive strength of 6.09 MPa, and thermal conductivity of 0.4794 W/m.K. Therefore, sucrose, as a porogen, facilitates the formation of the 3D network pore structure and the optimized pore size for PCCs using the Pickering emulsion technique method.

## 4. Conclusions

In this paper, PCCs with stable 3D microstructures were prepared by the Pickering emulsion technique, using sucrose as a porogen. The prepared PCCs precursor with sucrose content had the lowest initial Zeta potential, which was an important innovation to ensure stable suspension without acid or alkali additives to adjust the pH value. The microstructural and mechanical properties of PCCs could be controlled by varying O/S ratios (1–3), sintering temperature (1100–1400 °C), and sucrose content (10–40 wt%). The formation of the spherical pore structure resulted from the broken of oil bubbles. The formation of gradient pore structure was due to the formation of cordierite main phase and the concomitant consumption of sucrose during the sintering process. When the O/S ratio was 2, PCCs with a controlled pore structure was obtained at the sintering temperature of 1300 °C with cordierite as the primary phase. With a sucrose content of 30 wt.%, PCCs with a high solid content of 45 vol.% can be obtained, which had a porosity of 90.83%, a compressive strength of 6.09 MPa, and an optimized thermal conductivity of 0.4794 W/m.K. Our results showed that the sucrose not only acted as a solvent to increase solid content but also acted as a pH modifier to maintain precursor stability. PCCs prepared by the Pickering emulsion technique using sucrose as a porogen has potentially applicable value for the regulation of micron-scale and high-porosity ceramics.

## Data Availability

The relevant data and scripts used in the study are available when requested.
